# Influence of Secondary-Structure Folding on the Mutually Exclusive Folding Process of GL5/I27 Protein: Evidence from Molecular Dynamics Simulations

**DOI:** 10.3390/ijms17111962

**Published:** 2016-11-23

**Authors:** Qing Wang, Yan Wang, Guangju Chen

**Affiliations:** Key Laboratory of Theoretical and Computational Photochemistry, Ministry of Education, College of Chemistry, Beijing Normal University, Beijing 100875, China; wangqing0409@163.com

**Keywords:** molecular dynamics simulation, mutually exclusive folding process, GL5/I27 protein, secondary/tertiary-structure extending

## Abstract

Mutually exclusive folding proteins are a class of multidomain proteins in which the host domain remains folded while the guest domain is unfolded, and both domains achieve exchange of their folding status by a mutual exclusive folding (MEF) process. We carried out conventional and targeted molecular dynamics simulations for the mutually exclusive folding protein of GL5/I27 to address the MEF transition mechanisms. We constructed two starting models and two targeted models, i.e., the starting models GL5/I27-S and GL5/I27-ST in which the first model involves the host domain GL5 and the secondary-structure unfolded guest domain I27-S, while the second model involves the host domain GL5 and the secondary/tertiary-structure extending guest domain I27-ST, and the target models GL5-S/I27 and GL5-ST/I27 in which GL5-S and GL5-ST represent the secondary-structure unfolding and the secondary/tertiary-structure extending, respectively. We investigated four MEF transition processes from both starting models to both target models. Based on structural changes and the variations of the radius of gyration (*R*_g_) and the fractions of native contacts (*Q*), the formation of the secondary structure of the I27-guest domain induces significant extending of the GL5-host domain; but the primary shrinking of the tertiary structure of the I27-guest domain causes insignificant extending of the GL5-host domain during the processes. The results indicate that only formation of the secondary structure in the I27-guest domain provides the main driving force for the mutually exclusive folding/unfolding between the I27-guest and GL5-host domains. A special structure as an intermediate with both host and guest domains being folded at the same time was found, which was suggested by the experiment. The analysis of hydrogen bonds and correlation motions supported the studied transition mechanism with the dynamical “tug-of-war” phenomenon.

## 1. Introduction

The conformational transitions of some proteins play a crucial role in regulating protein function, and revealing fundamental biological processes of cell signaling and metabolism [1,2,3]. Many proteins built from structurally and functionally distinct domains display modular architecture, and accomplish widely diversified functions [4,5,6]. Individual domains in multidomain proteins are arranged in a way of connecting one domain followed by the next one in a contiguous order. Especially, a class of multidomain proteins, different from the general modular proteins, involves one guest domain inserted into the loop of another host protein [7,8]. An inserted guest domain disrupts the sequence continuity of a host protein to create a domain insertion protein. Such domain insertion proteins are usually used to generate a switching mechanism to regulate a variety of effector molecules, ligand binding, etc. [9,10,11]. Mutually exclusive folding protein as domain insertion protein or fusion protein serves to analyze the coupled folding–unfolding equilibrium through the conformational transition of antagonistic folding/unfolding [12,13,14,15]. Some of them can be also potentially regarded as a new class of cytotoxic proteins that can be activated by cell-specific effector molecules [15].

Mutually exclusive folding proteins are of particular interest in studies of protein folding/unfolding dynamics, and have been studied by many researchers [16,17,18,19,20,21,22,23,24]. To engineer such proteins, a guest domain is chosen from a special protein with the N–C terminal distance at least twice as long as the end-to-end distance of the loop in a host domain. Only one domain to remain folded with another one to be unfolded is allowed by topological constraints among the engineering of such proteins with structural incompatibility, which leads to the mutual exclusive folding (MEF) between the two domains [15]. Based on the engineered regulation, Stewart N. Loh and coworkers firstly constructed the domain-in-domain fused protein consisting of barnase (Bn) and ubiquitin (Ub) respectively as the host and guest domains. In such fused protein, the toxic activity of the host domain Bn is turned off when it is unfolded by the folding of the guest domain Ub [12,13,14,15]. Stewart N. Loh and coworkers in 2006 created the improved mutually exclusive folding protein, the GCN4 DNA binding domain as the guest and the ribonuclease barnase as the host, to regulate the RNase activity of the barnase domain through DNA binding to the GCN4 domain [25]. Hongbin Li and coworkers designed the novel mutually exclusive folding protein GL5/I27 consisting of the host protein GB1-L5 (GL5) from the B1 immunoglobulin-binding domain in streptococcal protein G [26,27] with the elongated loop, and the inserted guest protein I27w34f from the 27th immunoglobulin domain of the I-band of human cardiac titin [28,29,30]. In their work, the first kinetic evidence of the “tug-of-war” folding between the two I27w34f and GB1-L5 (GL5) domains, and the existence of a conformational equilibrium between the two folded conformations were reported by using single-molecule atomic force microscopy (AFM) techniques. It is also reported that the folding of the guest protein I27w34f can generate sufficient mechanical strain to unravel the host protein GL5, which cannot be found in traditional single-domain protein folding studies. By using fragments of GL5 as a model system, they demonstrated the proof-of-principle of using protein fragment reconstitution as a driving force to engineer self-assembling protein hydrogels [31,32]. Not only by using the two domains as the fundamental engineering module, the mutually exclusive folding principle has been expanded to construct a biosensor by using only a single folded domain. Such a biosensor is a protein containing duplicated segments in which only one of the two fragments at a time is incorporated in the folded protein. Stratton et al. developed the alternate frame folding (AFF) strategy from a wild-type fold (N) to its circularly-permuted fold (N’) to build a switchable protein based on conformational changes [33,34,35,36,37,38]. Although MEF and AFF are incorporated into the folding-unfolding switch design, experimental studies on their switch mechanisms and the details of conformational variations at atomic level are limited so far. Molecular dynamics (MD) simulation provides powerful methodology to independently monitor the individual protein domains in the mutually exclusive and alternate frame folding processes, which would be helpful for experimental studies. MD simulations represent a possibility to describe structure and dynamics of macromolecules at atomistic resolution simultaneously [39]. Therefore, MD simulations have become an invaluable tool for the investigation of biological processes. Initially mainly used in theoretical chemistry, MD tools are nowadays extensively used by scientists from a broad range of research areas [40,41]. Lillian T. Chong and coworkers theoretically explored the kinetics of mutually exclusive folding by determining the effect of linker length and ligand binding on rates of unfolding and refolding of each protein domain for the barnase-ubiquitin fusion protein switch by using molecular dynamics simulations. Their results qualitatively agreed with analytical ultracentrifugation experiments [42]. Moreover, the transition mechanism of N’ and N conformations of the alternate frame folding calbindin-D_9k_ protein were investigated by using the targeted molecular dynamics simulations in our previous work [43]. However, theoretical investigations on the dynamical transition mechanism and structural changes of mutually exclusive folding process for the GL5/I27 fusion protein have not yet been studied in detail at the atomic level.

In order to explore the mutually exclusive folding mechanism at the atomic level, we carried out conventional molecular dynamics (CMD) and targeted molecular dynamics (TMD) simulations to investigate mutually exclusive folding transition processes for the GL5/I27 fusion protein. Our main objectives were (1) to address the structural property of transition processes with different unfolding degrees of GL5 and I27 domains; (2) to investigate the transition regularity for these processes by analyzing variation of the radius of gyration (*R*_g_) and the fraction of native contacts (*Q*); (3) to explore the nature of the main driving force for mutually exclusive folding/unfolding processes.

## 2. Results

### 2.1. Initial Structure Construction

**Figure 1:**
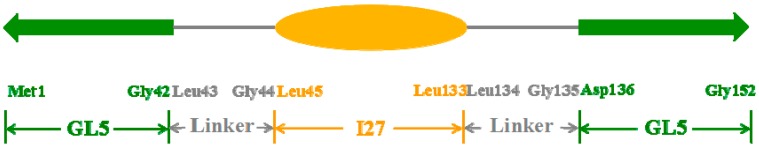
The amino acid sequences of GL5/I27 mutually exclusive protein. Amino acids are numbered according to the Nuclear Magnetic Resonance (NMR) structures of GB1 (PDB:2GB1) and I27 (PDB:1TIT) proteins. The subsequences of GL5-host domain, I27-guest domain and linkers in GL5/I27 protein are colored in green, orange, and gray, respectively.

The normal mutually exclusive folding protein contains a guest protein inserting into the certain loop region of a host protein with folded/unfolded structures. Based on the previous experimental studies, the mutually exclusive folding protein models, GL5*_i_*/I27*_i_* (*i* = S or ST), with the different unfolding degrees of the I27-guest domain or the GL5-host domain were designed from the Nuclear Magnetic Resonance (NMR) structures of I27 (PDB:1TIT) and GB1 (PDB:2GB1) proteins [27,28]. The GL5-host domain (61 residues) was designed from the GB1 protein (56 residues) with the elongate loop by five additional residues Gly-Gly-Gly-Leu-Gly inserting to the middle of Val39 and Leu40 of GB1 protein. Especially, for constructing the whole protein model, the two additional residues, Leu and Gly, in the GL5-host domain were duplicated for building the non-palindromic A*va*I linker site in order to be inserted by the I27-guest domain (89 residues). Therefore, the constructed mutually exclusive folding protein model consists of the GL5-host domain (Met1–Gly42, Asp136–Glu152), the I27-guest domain (Leu45–Leu133), and the linker (Leu43, Gly44, Leu134, and Gly135) between the GL5-host domain and I27-guest domain (see Figure 1). The two models used in the MD simulations correspond to the two structures of the GL5/I27 protein with different unfolding degrees of the I27-guest domain, i.e., the secondary-structure unfolding and the secondary/tertiary-structure extending in the I27-guest domain, respectively assigned as GL5/I27-S and GL5/I27-ST models. The folded coordinates of Met1–Val39, Asp136–Gly152, and Leu45–Leu133 sequences in the GL5-host and I27-guest domains for the GL5/I27-S and GL5/I27-ST models were taken from the NMR structures of the GB1 (PDB:2GB1) and I27 (PDB:1TIT) proteins. Then the total coordinates of the folded/unfolded regions for such sequences, the five additional residues of Leu40–Gly44 in the GL5-host domain and the linker of Leu134, Gly135 were built and modified by using the loop search method in the Swiss-PdbViewer (also known as DeepView, http://spdbv.vital-it.ch/) and the Discovery Studio visualizer (http://accelrys.com/). The details of the model construction are given in the Supplementary Materials. Such modified coordinates with the NMR structure of the GL5-host domain containing the additional residues, the constructed structures of the unfolded I27-guest domain and the linker form the initial structures of two GL5/I27-S and GL5/I27-ST models as the GL5/I27 proteins for the MD simulations. Similarly, the other two models of GL5-S/I27 and GL5-ST/I27 corresponding to the extended GL5-host domain with the different unfolding degrees, i.e., the secondary-structure unfolding and the full extending in the GL5-host domain, were built by using the same method. Each of the models was explicitly solvated by using the TIP3P water potential inside an orthorhombic box of water molecules with a minimum solute-wall distance of 8 Å.

### 2.2. Structures of the Mutually Exclusive Folding Models of GL5_i_/I27_i_

**Figure 2:**
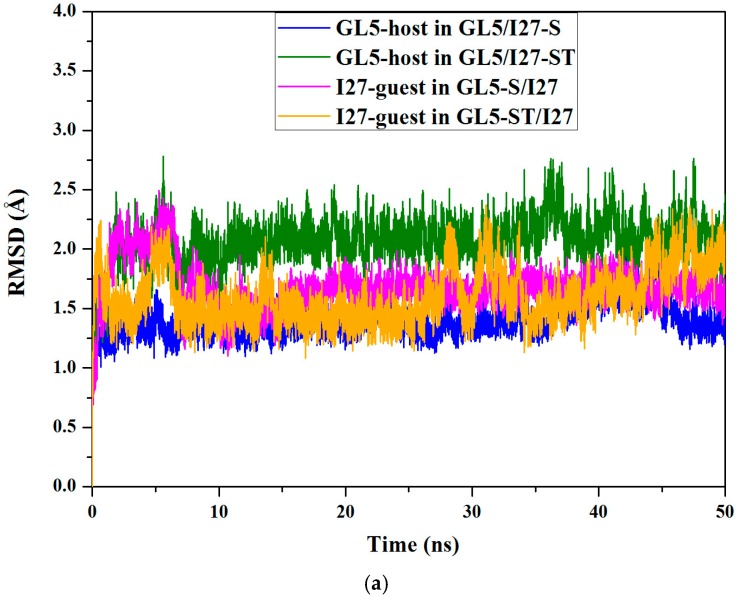

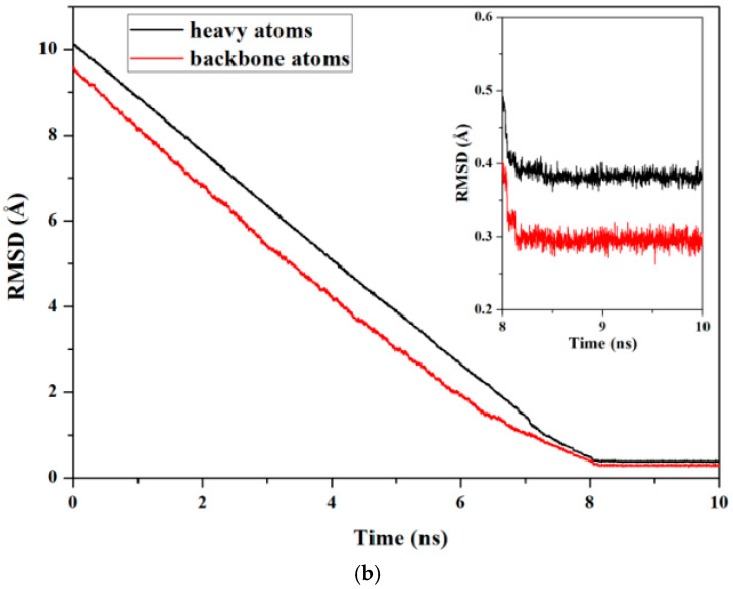
Root mean square deviation (RMSD) values: (**a**) all backbone atoms of the GL5-host domain in the GL5/I27-S and GL5/I27-ST models, and the I27-guest domain in the GL5-S/I27 and GL5-ST/I27 models from the conventional molecular dynamics (CMD) simulations; (**b**) all backbone atoms and all heavy atoms of the whole protein from the targeted molecular dynamics (TMD) simulation of transition of GL5/I27-S to GL5-ST/I27.

For constructing a special class of the mutually exclusive folding proteins GL5/I27 with the guest domain I27 inserting into the host domain GL5, four protein models of GL5/I27-S, GL5-S/I27, GL5/I27-ST, and GL5-ST/I27 with the different unfolding degrees of the guest and host domains were constructed. Based on these constructed models, the 50-ns CMD simulations were first carried out to understand the stability of these structures. The root mean square deviation (RMSD) values of all backbone atoms relative to the NMR data for the native domains except for the atoms in the loop regions over the corresponding trajectories for the four models were examined to determine the system equilibriums, and are shown in Figure 2a. The calculated conformational energies for GL5/I27-S and GL5-ST/I27 models were also analyzed and are shown in Figure S1a,b of the Supplementary Materials. As illustrated in Figure 2a and Figure S1a,b, each of the systems reached equilibrium after 20 ns with stable energies. Therefore, the trajectory analysis of the systems yielded the equilibrated conformations between 40 ns and 50 ns simulation times, recording 5000 snapshots at every 2 ps time-interval of each trajectory. 

**Figure 3:**
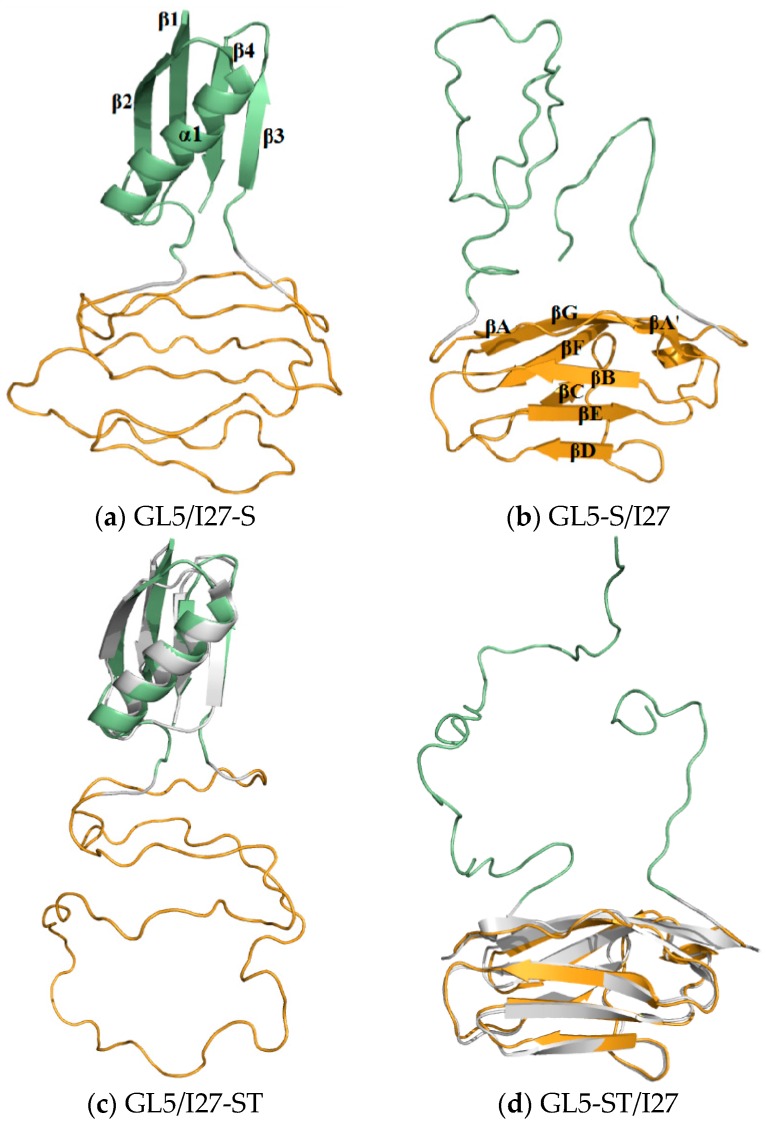
Three-dimensional average structures for (**a**,**c**) GL5/I27-S and GL5-S/I27 states with the secondary-structure unfolding of I27-guest and GL5-host domains, respectively; (**b**,**d**) GL5/I27-ST and GL5-ST/I27 states with the secondary/tertiary-structure extending of I27-guest and GL5-host domains and the superpositions from the NMR I27 and GB1 proteins (PDB-ID 1TIT and 2GB1), respectively. The components of I27-guest domains, GL5-host domains, linkers and the superpositions are colored in orange, olive, gray, and white, respectively.

The four corresponding average structures of GL5/I27-S, GL5-S/I27, GL5/I27-ST, and GL5-ST/I27 models are shown in Figure 3a–d with the corresponding superpositions of the NMR structures of the GB1 (PDB:2GB1) and I27 (PDB:1TIT) proteins. According to the analysis on the simulated structures, the simulated native GL5-host and I27-guest domains in the four models reflect the characteristics of the experimental NMR structures very well. Namely, the native GL5-host domain for the GL5/I27-S and GL5/I27-ST models in Figure 3a,c includes four β strands, i.e., β1(Met1–Asn8), β2 (Leu12–Ala20), β3 (Gly138–Gly142), β4 (Tyr146–Thr151), and a helix of α1 (Ala23–Asn37) with the connecting loops of loop_1–2_ (Gly9–Thr11), loop_2–α_ (Val21–Asp22), loop_α_ (Gly38–Gly44), loop_3_ (Asp136, Gly137), and loop_3–4_ (Asp143–Thr145). Its structure presents one layer consisting of four parallel strands in the order of β2–β1–β4–β3, and the other layer consisting of the α1 helix [27]. As shown in Figure 3b,d, the native I27-guest domain includes seven β strands, i.e., βA’/βA (Val48–Pro51/Val55–Val57), βB (Thr62–Glu68), βC (Gln77–Lys79), βD (Cys91–Asp96), βE (Lys99–Hie105), βF (Gly113–Ala119), βG (Ala122–Val130) in the order of βA’/βA–βB–βC–βD–βE–βF–βG, which present a cage structure, and several loops ofloop_A_ (Leu45–Gly54), loop_A–B_ (Phe58–Glu61), loop_B–C_ (Leu69–Gly76), loop_C–D_ (Leu80–Asp90), loop_D–E_ (Asp96–Lys98), loop_E–F_ (Asn106–Thr112), loop_F–G_ (Ala120, Asn121), and loop_G_ (Lys131–Leu133) [28]. The extended I27-guest and GL5-host domains present the secondary-structure unfolding for the GL5/I27-S and GL5-S/I27 models in Figure 3a,b, and the secondary/tertiary-structure extending/the full extending for the GL5/I27-ST and GL5-ST/I27 models in Figure 3c,d, respectively. 

### 2.3. Characteristics of the Induced Full Extending Processes of the Mutually Exclusive Folding Models GL5_i_/I27_i_

**Figure 4:**
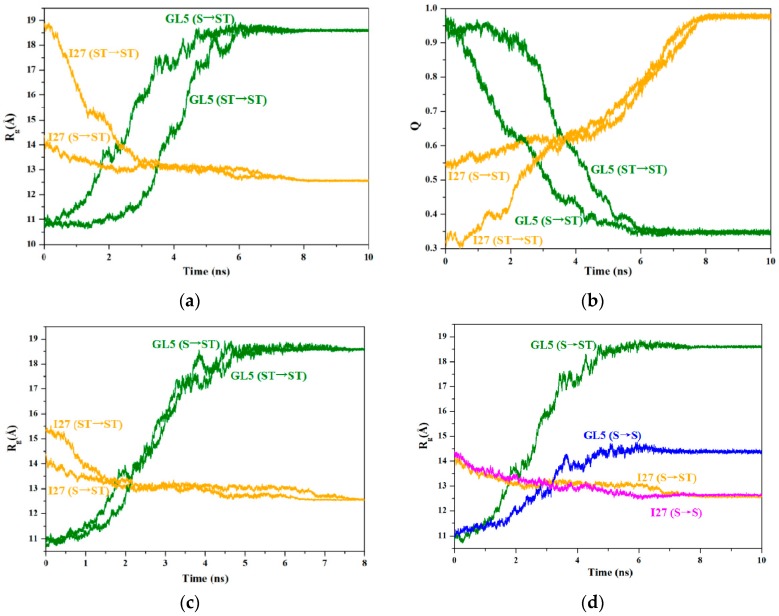
Variations for (**a**) the radius of gyration (*R*_g_) values and (**b**) the fractions of native contacts (*Q*) values of I27-guest (orange) and GL5-host domains (olive) for GL5/I27-S and GL5/I27-ST models respectively to GL5-ST/I27 model (i.e., S→ST and ST→ST); (**c**) superposition of *R*_g_ variations at TMD time of 0 ns for GL5/I27-S to GL5-ST/I27 over at 1.3 ns for GL5/I27-ST to GL5-ST/I27; (**d**) variations of *R*_g_ of I27-guest (orange and magenta) and GL5-host domains (olive and blue) for GL5/I27-S model to GL5-ST/I27 and GL5-S/I27 models (i.e., S→ST and S→S), respectively.

To address the mutually exclusive folding characteristics for the full extending of the GL5-host domain, we successfully gained the mutually exclusive folding processes from the starting GL5/I27-S and GL5/I27-ST models with the secondary-structure unfolding and the secondary/tertiary-structure extending of the I27-guest domains respectively to the target GL5-ST/I27 model with the full extending of the GL5-host domain by performing the 10-ns TMD simulations with the bias force constant of 3.0 kcal/(mol·Å^2^). The plots for the RMSDs of all backbone and heavy atoms in the TMD-simulated structures relative to the corresponding target states are shown in Figure 2b for GL5/I27-S to GL5-ST/I27 models and Figure S2a of the Supplementary Materials for GL5/I27-ST to GL5-ST/I27, respectively. These results reveal that all backbone and heavy atoms in four TMD simulations reached the target conformations with an accuracy of <1 Å within 8 ns. Generally, the radius of gyration (*R*_g_) and the fraction of native contacts (*Q*) in a protein are respectively used to measure the degrees of the unfolding and similarity of a protein to its native state (defined as *Q* = 0 or 1 at a fully unfolded state or a native state) [44,45,46,47,48]. To explore the details of the dynamic process during the mutually exclusive folding, the variations of the radius of gyration (*R*_g_) and the fraction of native contacts (*Q*) for both I27-guest and GL5-host domains along the two TMD simulations of both GL5/I27-S and GL5/I27-ST models to GL5-ST/I27 model were extracted from the corresponding trajectories by using the PTRAJ module of AMBER 9 program, and are shown in Figure 4a,b, respectively. The computational details are given in the Supplementary Materials. 

**Figure 5:**
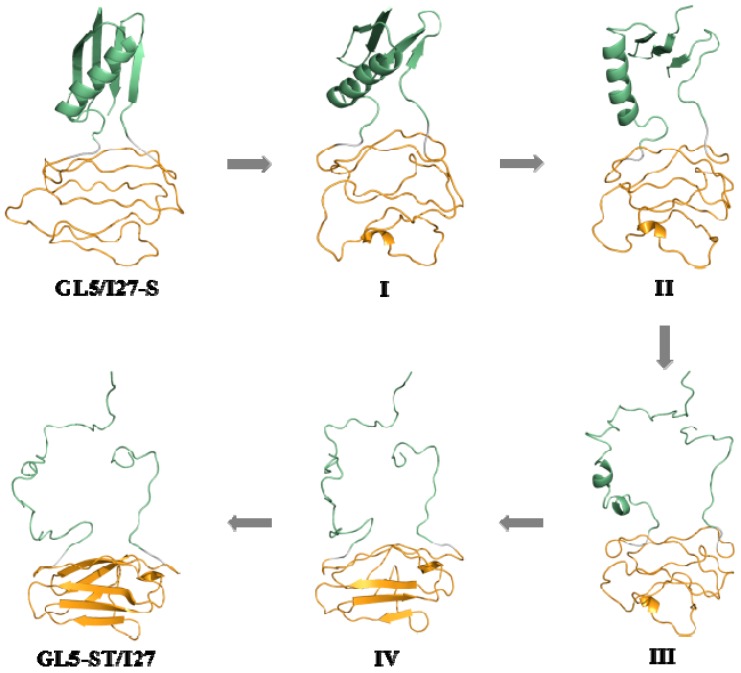
The three-dimensional average structures for GL5/I27-S, GL5-ST/I27 states and I, II, III, IV conformations for the transition of GL5/I27-S to GL5-ST/I27 via I, II, III and IV. The components of I27-guest domains, GL5-host domains and linkers are colored in orange, olive and gray, respectively.

For the process of the GL5/I27-S model with the secondary-structure unfolding of the I27-guest domain to the GL5-ST/I27 model with the full extending of the GL5-host domain, the time-average structures of the starting state (GL5/I27-S), the four transition conformations (I (~1.1 ns), II (~2.2 ns), III (~3.7 ns), IV (~6.5 ns)) and the target state (GL5-ST/I27) were extracted along with the CMD/TMD simulation, and are shown in Figure 5. It is can be seen that the structural changes mainly involve the curliness of amino acids followed by the formation of the secondary structure of seven strands in the I27-guest domain along with the induced extending of the GL5-host domain with the deformation of the four-strands layer followed by the unfolding of the α1 helix. Based on the analysis of the conformations and the radius of gyration (*R*_g_) in Figure 4a, the *R*_g_ values of the I27-guest domain decline slowly from 14.4 Å at 0 ns to 12.6 Å at ~7 ns due to the formation of secondary structures; simultaneously, those of the GL5-host domain increase rapidly from 10.8 Å at 0 ns to 18.6 Å at ~6 ns during this process. The large deviation of *ΔR*_g_ of 7.8 Å reflects the significant extending of the GL5-host domain induced by the slow formation of secondary structure of the I27-guest domain with a small deviation of 1.8 Å. Furthermore, the uniform variation of *R*_g_ values for the two domains reveals qualitatively the complicated dynamic process of “tug-of-war” event under the mutually exclusive folding, suggested by the experiment [30]. Furthermore, the expected similar variations of the fractions of native contacts *Q* in Figure 4b for this process show the increase of *Q* values of the I27-guest domain from 0.54 at GL5/I27-S to 0.98 at GL5-ST/I27, but the decrease of that of the GL5-host domain from 0.97 to 0.34, which reflects the significant formation of the native I27-guest domain and the approximately full extending of the GL5-host domain due to the corresponding *Q* values approaching 1 and deviating from 1. The results illustrated that the formation of only secondary structures in the I27-guest domain may cause full extending of the GL5-host domain. Moreover, the analysis of correlation network for the dynamical mutually exclusive folding revealed the structural characteristics of the native guest and host domains, and the mutually exclusive communication between the formation of the I27-guest domain and the extending of the GL5-host domain during the transition process.

To address how the extended size of the I27-guest domain influences the full extending of the GL5-host domain, the time-average structures of the starting state (GL5/I27-ST) with the secondary/tertiary-structure extending of the I27-guest domain, the five transition conformations (I’ (~1.1 ns), II’ (~2.2 ns), III’ (~3.7 ns), IV’ (~4.8 ns), V’ (~6.8 ns)) and the target state (GL5-ST/I27) were extracted along with the TMD simulation of GL5/I27-ST to GL5-ST/I27, and are shown in Figure S3 of the Supplementary Materials. The structural changes in the transition of GL5/I27-ST to GL5-ST/I27 involve the primary shrinking of the tertiary structure of the I27-guest domain followed by the similar curliness of amino acids to form the secondary structure along with the induced extending of the GL5-host domain. Based on these structural characteristics and the analysis of *R*_g_ variations in Figure 4a for GL5/I27-ST to GL5-ST/I27, the *R*_g_ values of the I27-guest domain decline rapidly from 18.9 Å at 0 ns to 12.6 Å at ~6 ns due to the large size of the I27-guest domain in the starting GL5/I27-ST model and the primary shrinking of the tertiary structure followed by the secondary-structure formation, which is different from 14.4 Å at 0 ns in the starting GL5/I27-S model with the small size of the I27-guest domain, discussed above. However, those of the GL5-host domain remain at 10.8 Å in the native GL5 state during the first 1.3 ns, then increase rapidly from 10.8 Å to 18.6 Å during the remaining TMD simulation time. These data reflect that the primary decrease of *R*_g_ values in the I27-guest domain causes insignificant extending of the GL5-host domain due to the shrinking of only tertiary structure in the I27-guest domain. The further decrease of *R*_g_ values in the I27-guest domain induces significant extending of the GL5-host domain. Especially, it can be seen that the primary tertiary-structure shrinking of the I27-guest domain does not cause extending of the GL5-host domain during the period of the *R*_g_ values of the I27-guest domain decreasing from 18.9 Å at 0 ns to 15 Å at 1.3 ns. That is, the full extending of the GL5-host domain occurs only after the *R*_g_ values of the I27-guest domain decrease from 15 Å at 1.3 ns to 12.6 Å at 6 ns. It should be noted that the size of I27-guest domain *R*_g_ = 15 Å in the GL5/I27-ST to GL5-ST/I27 process almost equals that of *R*_g_ = 14.4 Å in the GL5/I27-S to GL5-ST/I27 process discussed above. The results indicate that the formation of the secondary structure provides the main driving force for extending the native GL5-host domain. The larger I27-guest domain with the secondary/tertiary-structure extending promotes insignificantly the extending of the GL5-host domain. That is, the primary tertiary-structure shrinking of the I27-guest domain has not yet induced the extending of the GL5-host domain. To further illustrate the nature of the driving force of secondary-structure folding, the superposition of variations of the *R*_g_ values at the TMD time of 0 ns for the process of GL5/I27-S to GL5-ST/I27 over that at 1.3 ns for GL5/I27-ST to GL5-ST/I27 is shown in Figure 4c, which reveals that the mutually exclusive folding process of GL5/I27-ST to GL5-ST/I27, starting from the large extended I27-guest domain with the same unfolding velocity during the simulation time of 1.3–5.8 ns, reproduces the process of GL5/I27-S to GL5-ST/I27 from the small extended I27-guest domain during 0–4.5 ns. Moreover, the variation of *Q* for GL5/I27-ST to GL5-ST/I27 in Figure 4b reflects the similar variation tendency with *R*_g_, that is, *Q* values of the I27-guest domain increase from 0.32 at GL5/I27-ST to 0.98 at GL5-ST/I27, while that of the GL5-host domain decrease from 0.98 to 0.35. Especially, *Q* = 0.32 at the GL5/I27-ST to GL5-ST/I27 process reveals the larger I27-guest domain with the secondary/tertiary-structure extending, which is compared to the *Q* = 0.54 in GL5/I27-S to GL5-ST/I27 process. Summarizing, the investigation of the two processes from the starting structures with the different guest-domain sizes to the full extending of the host domain reveals that the secondary-structure folding of the guest domain is the driving force for the mutually exclusive folding process.

### 2.4. Characteristics of the Induced Secondary-Structure Unfolding Processes of the Mutually Exclusive Folding Models GL5_i_/I27_i_

To address further the effect of the extending size of the I27-guest domain on the secondary-structure unfolding of the GL5-host domain, the TMD simulations from the starting GL5/I27-S and GL5/I27-ST models, that are the same models in the processes discussed above, to another target GL5-S/I27 model with the secondary-structure unfolding of the GL5-host domain were performed, and similar mutually exclusive folding processes were also successfully attained. The plots for the RMSDs of all backbone and heavy atoms are shown in Figure S2b,c of the Supplementary Materials for GL5/I27-S to GL5-S/I27 and GL5/I27-ST to GL5-S/I27, respectively. The variations of the radius of gyration (*R*_g_) and the fraction of native contacts (*Q*) for both the I27-guest and GL5-host domains along the two TMD simulations were also extracted from the corresponding trajectories, and are shown in Figures S4a,b of the Supplementary Materials, respectively. The variation characteristics of *R*_g_ for the process of GL5/I27-S to GL5-S/I27 in Figure S4a predict the slow uniform decrease of *ΔR*_g_ = 1.8 Å for the I27-guest domain, and the increase of *ΔR*_g_ = 3.4 Å for the GL5-host domain, in which the increase of the *ΔR*_g_ values in the GL5-host domain reflects only the secondary-structure extending of the GL5-host domain. However, the variation of the *R*_g_ values for the process of GL5/I27-ST to GL5-S/I27 shows the rapid decrease of *ΔR*_g_ = 6.2 Å for the I27-guest domain, and the steady fluctuation followed by slow increase of *ΔR*_g_ = 3.4 Å for the GL5-host domain. The *R*_g_ decrease in the I27-guest domain and its steady fluctuation in the GL5-host domain predict that the primary shrinking of only the tertiary structure in the I27-guest domain causes insignificant extending of the GL5-host domain. However, the continuous *R*_g_ decrease in the I27-guest domain and the following increase in the GL5-host domain reflect that the secondary-structure formation in the guest domain induces rapid extending of the host domain. These results still support the crucial function of the secondary-structure formation in the guest domain for the mutually exclusive folding process. It is worth noting that a special structure at the simulation time of ~3.3 ns during the process of GL5/I27-S to GL5-S/I27 was found with both I27-guest and GL5-host domains being folded at the same time, which has been suggested by the experiment as an intermediate occurring on rare occasions [30]. The corresponding average structure is shown in Figure S5 of the Supplementary Materials, which presents the partial folded regions of β1~β3 strands and α1 helix in the GL5-host domain, and βC, βF strands in the I27-guest domain.

### 2.5. Comparison between the Induced Full Extending and Secondary-Structure Unfolding Processes

To address the mutually exclusive folding characteristics from the starting state with the small size of I27-guest domain involving only the secondary-structure unfolding to the target states with the large and small sizes of GL5-host domains, the comparison between the two processes of GL5/I27-S to GL5-ST/I27 and GL5/I27-S to GL5-S/I27 was analyzed, and the corresponding variations of *R*_g_ for both I27-guest and GL5-host domains are shown in Figure 4d. The expected variation rates of *R*_g_ of the GL5-host domains for both processes of GL5/I27-S to GL5-ST/I27 and GL5/I27-S to GL5-S/I27 are about 0.98 and 0.43 Å/ns, respectively, during the assigned simulation time of 8 ns, compared with actual variation rates of 1.56 and 0.68 Å/ns during the actual simulation time of 5 ns. The deviation between the expected and actual rates of *R*_g_ of the GL5-host domains for the process of GL5/I27-S to GL5-ST/I27 is larger by 0.33 Å/ns than that for GL5/I27-S to GL5-S/I27, which indicates that the folding for GL5/I27-S to GL5-ST/I27 with full extending of the GL5-host domain is faster than that for GL5/I27-S to GL5-S/I27 with secondary-structure unfolding. Moreover, the cross site of *R*_g_ curves of the GL5-host and I27-guest domains at *R*_g_ = 13.1 Å occurs firstly at the simulation time of 1.8 ns for GL5/I27-S to GL5-ST/I27 over that at the simulation time of 3.2 ns for GL5/I27-S to GL5-S/I27 due to the rapid variation rate for GL5/I27-S to GL5-ST/I27, which also supports the rapid folding of GL5/I27-S to GL5-ST/I27. The results indicate that the secondary-structure formation in the guest domain could generate enough driving force to induce full extending of the host domain.

## 3. Discussion

### 3.1. Analysis of the Structural Changes for the Induced Full Extending Processes

**Figure 6:**
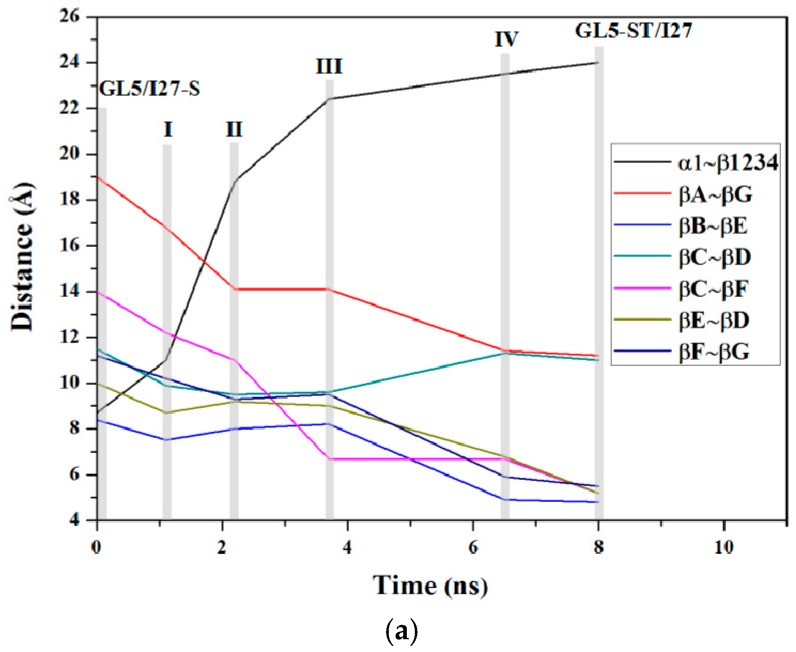

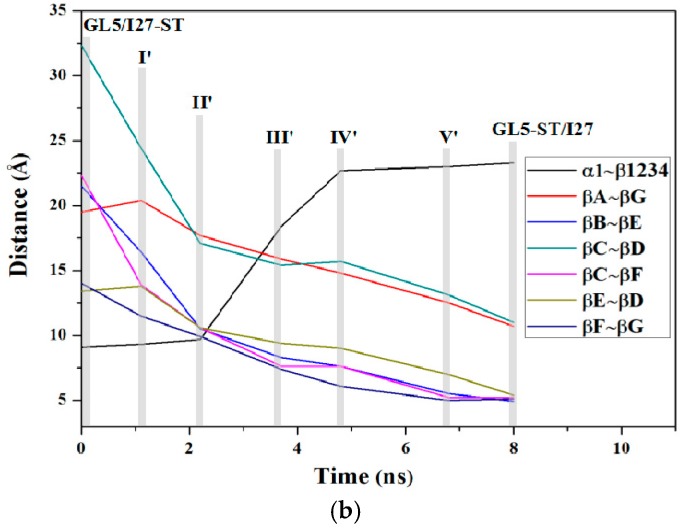
Schemes of mass center distances between the adjacent β-strands in the I27-guest domain along with the TMD simulations of (**a**) GL5/I27-S to GL5-ST/I27 and (**b**) GL5/I27-ST to GL5-ST/I27 with the grey areas denoting GL5/I27-S, GL5-ST/I27 states and I, II, III, IV conformations, and GL5/I27-ST, GL5-ST/I27 states and I’, II’, III’, IV’, V’ conformations, respectively.

The details of the structural changes for the starting state (GL5/I27-S), the four transition conformations (I (~1.1 ns), II (~2.2 ns), III (~3.7 ns), IV (~6.5 ns)) and the target state (GL5-ST/I27) in the mutually exclusive folding pathway from the GL5/I27-S model with the secondary-structure unfolding I27-guest domain to the GL5-ST/I27 model with the full extended GL5-host domain in Figure 4 were analyzed. The occurrences of all possible hydrogen bonds are shown in Table 1 and Table 2, and the mass center distances between the adjacent β-strands of the I27-guest domain are shown in Figure 6a along with the corresponding CMD/TMD simulation for these conformations by using the PTRAJ module of the AMBER 9 program. The computational details are given in the Supplementary Materials. It can be seen that the structural changes in the process mainly involve the formation of the secondary-structure of the I27-guest domain followed by the formation of the I27-guest domain with the formational order of βB, βE, βD, then βA’/βA, βC, βF, βG strands along with the induced extending of the GL5-host domain with the deformation of the four-strands layer followed by the unfolding of the α1 helix. Namely, in the first step from GL5/I27-S to I to II with the slow *R*_g_ declining of the I27-guest domain and the rapid *R*_g_ increasing of the GL5-host domain, the rearrangement of the β sheets in the I27-guest domain causes tightness of the cage structure with decreases of the mass center distances between βA/βF, βC and βG, βF strands and the formation of several hydrogen bonds. Simultaneously, the GL5-host domain deforms at I, then the β1–β4, β1–β2, and β3–β4 sheets are partially unfolded at II, which causes decrease of the relative percentages of the total hydrogen bond occupancies of the β sheets occupancies by 42.6% from GL5/I27-S to II—the computational details are given in the Supplementary Materials. For example, the distance between the βA and βG strands in the I27-guest domain decreases from ~19.0 Å in the GL5/I27-S model to ~16.8 Å at I, then to ~14.4 Å at II (see Figure 6a). In the second step from II to III then to IV, the continuous tightness of the cage structure of the I27-guest domain causes the formations of βB, βD, βE strands at III and βB–βE, βE–βD sheets at IV with considerable decreases of the distances between βA’/βA/βF, βB, βC, βE and βG, βE, βF, βD strands, and the increase of the relative percentages of the total hydrogen bond occupancies from 17.78% at II to 23.58% at III then to 59.55% at IV (see Figure 6a and Table 1). Simultaneously, the β sheets and α1 helix in the GL5-host domain gradually unfold from II to IV with decrease of the relative percentages of the total hydrogen bond occupancies from 58.5% at II to 21.8% at III then to 0% at IV. For example, the distances between the βC and βF strands, and between βE and βD strands in the I27-guest domain decrease by ~4.3 Å and ~2.4 Å from II to IV (see Figure 6a). These results indicate that the tightness of the cage structure of the I27-guest domain, i.e., the formation of secondary structures of βA’/βA–βB, βB–βE, βE–βD sheets, causes the unfolding of β1–β2, β1–β4, β3–β4 sheets and α1 helix resulting in the extending of the GL5-host domain, which further supports the important role of the secondary structural rearrangement of the I27-guest domain in the extending of the GL5-host domain. In the last step from IV to GL5-ST/I27 model, the formations of βA’/βA–βG, βC–βF and βF–βG sheets of the I27-guest domain with the complete cage structure reproduce the structure of native I27 state. However, the GL5-host domain completes its full extending procedure. Summarily, such analysis of the structural changes in the full extending processes of GL5/I27-S to GL5-ST/I27 illustrates that the mutual correspondence of the secondary structural formation in the guest domain to the extending of the host domain exhibits the dynamic process of “tug-of-war” event.

To compare the two full extending processes of GL5/I27-S and GL5/I27-ST to GL5-ST/I27, the details of structural changes for the starting state (GL5/I27-ST), the four transition conformations (I’ (~1.1 ns), II’ (~2.2 ns), III’ (~3.7 ns), IV’ (~4.8 ns), V’(~6.8 ns)) and the target state (GL5-ST/I27) in the pathway from the GL5/I27-ST model with the larger extending of the I27-guest domain to the GL5-ST/I27 model with the full extended GL5-host domain were also analyzed (see Figure S3). The occurrences of all possible hydrogen bonds for these models, and the mass center distances between the adjacent β-strands of the I27-guest domain along with the corresponding TMD simulation are shown in Tables S1 and S2 of the Supplementary Materials and Figure 6b. The structural changes in the full extending processes of GL5/I27-ST to GL5-ST/I27 involve primary shrinking of the tertiary structure of the I27-guest domain, and a similar mechanism of mutually exclusive folding for GL5/I27-S to GL5-ST/I27. From GL5/I27-ST to I’ then to II’ conformations with rapid *R*_g_ declining of the I27-guest domain and *R*_g_ invariableness of the GL5-host domain, the large extending I27-guest domain gradually shrinks with significant decreases of the mass center distances between βB, βC, βF and βE, βD, βG strands from ~21.5 Å, ~32.3 Å, ~14.0 Å at GL5/I27-ST to ~10.5 Å, ~17.1 Å, ~9.9 Å at II’, respectively, which mainly contributes to the rapid *R*_g_ declining. Especially, the large shrinking of I27-guest domain causes insignificant extending of the GL5-host domain verified by its *R*_g_ invariableness from GL5/I27-ST to II’. Furthermore, the formation of few inner-hydrogen bonds in the secondary structure of the I27-guest domain predicts its rearrangement of the tertiary structure (see Table S2). For the remaining process from II’, III’, IV’, V’ to GL5-ST/I27 model, the similar structural changes in the process of GL5/I27-S to GL5-ST/I27 for three steps discussed above were found due to the structure of II’ presenting the main conformational characteristics of the GL5/I27-S model. That is, larger extending of the I27-guest domain with secondary/tertiary-structure extending in the GL5/I27-ST model firstly shrinks to the size of the I27-guest domain in GL5/I27-S model with the secondary-structure unfolding, then the formation of the βA’/βA, βB, βE, βG strands followed by the formation of the βC, βD, βF strands with cage structure in the I27-guest domain causes the unfolding of β1–β2, β1–β4, β3–β4 sheets and α1 helix, i.e., the full extending of the GL5-host domain (see Figure S3). Therefore, these results also reveal that the secondary structural formation in the I27-guest domain serves as the main force to drive the full extending of the GL5-host domain from its native structure, further to trigger the “tug-of-war” event between the guest and host domains of the mutually exclusive folding protein.

### 3.2. Mutually Exclusive Correlation Network in the Induced Full Extending Process

**Figure 7:**
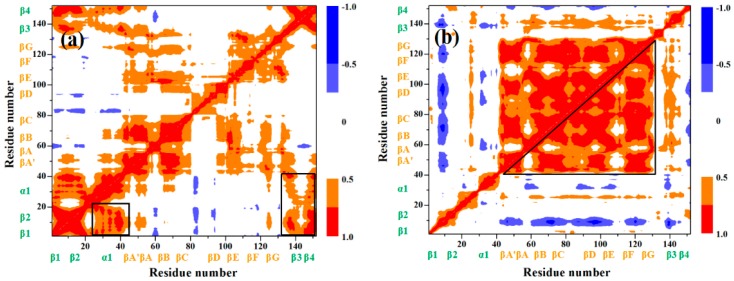

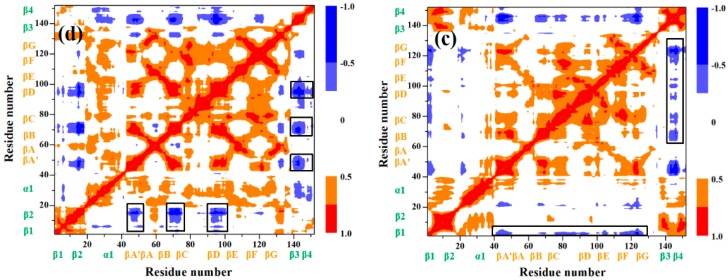
Dynamical cross-correlation maps for (**a**) the GL5/I27-S and (**b**) the GL5-ST/I27 models; (**c**) the II and (**d**) IV conformations with the key subregions squared and triangulated in black for the transition of GL5/I27-S to GL5-ST/I27. High motion correlations and anticorrelations between the residues are colored in red and blue.

To explore the mutually exclusive communications of each residue in the I27-guest and GL5-host domains, we analyzed the motion correlations of all Cα atoms for the GL5/I27-S, GL5-ST/I27 models, and II, IV conformations in the process of the GL5/I27-S to GL5-ST/I27 models extracting from the simulation trajectories, which are displayed in Figure 7a–d, respectively—the computational details are given in the Supplementary Materials. These maps show high motion correlations (red) and anticorrelations (blue) between the residues. As expected, the motions of the β1–β2, β1–β4, β3–β4 sheets and the α1 helix of the GL5-host domain in the GL5/I27-S model significantly correlate with each other represented by the black squares in Figure 7a, which indicates the characteristics of the native GL5 state consisted of two layers, four β-strands and one α helix. For the GL5-ST/I27 model, the significant motion correlations among the βA–βG strands in the I27-guest domain were found (represented by the black square in Figure 7b), which indicates the cage structure of the native I27 state. However, the non-correlations of the β1–β2, β1–β4, β3–β4 sheets and the α1 helix in the GL5-host domain show the secondary structural unfolding, resulting in the full extending of the host domain. Figure 7c,d for II and IV conformations predict the increase of correlations among the βA–βG strands in the I27-guest domain and the decrease of that among the β1–β2, β1–β4, β3–β4 sheets and the α1 helix in the GL5-host domain due to the gradual formation of the secondary structures of the I27-guest domain corresponding to the gradual extending of the GL5-host domain along with such a mutually exclusive folding process. Especially, the anticorrelation between the βB, βC, βD, βF, βG strands of the I27-guest domain and the β1–β2, β3–β4 sheets of the GL5-host domain at the II conformation predicts the rearrangement of these β strands inducing partial unfolding of β sheets in the GL5-host domain (see Figure 7c). In the IV conformation, the anticorrelations between the βA’/βA, βB, βD, βE strands of the I27-guest domain and the β1–β2, β1–β4, β3–β4 sheets of the GL5-host domain support the formation of βA’/βA–βB, βB–βE, βE–βD sheets and the extending of the β sheets and α1 helix (see Figure 7d). It can be seen that the correlation characteristics in these conformations well illustrate the mutually exclusive communication between the formation of the β sheets of the I27-guest domain and the extending of the GL5-host domain, i.e., the dynamical “tug-of-war” event.

## 4. Methodology

### 4.1. Conventional Molecular Dynamics Simulation

All conventional molecular dynamics (CMD) simulations for the four models were performed by using the AMBER 9 package [49] together with ff03 all atom force field parameters [50,51,52]. The protocol for all CMD simulations for the GL5/I27-S, GL5/I27-ST, GL5-S/I27, and GL5-ST/I27 models contains several steps: i.e., the models were energetically minimized to remove unfavorable contacts; each energy-minimized model was heated from 0 to 300 K, and the unrestrained equilibration of 200 ps with constant pressure and temperature conditions was carried out for each model; finally, conventional molecular dynamics (CMD) runs of 50 ns for these models were carried out by following the same protocol. The computational details of the CMD procedure are given in the Supplementary Materials.

### 4.2. Targeted Molecular Dynamics Simulation

Generally, the structure transitions are beyond the reach of conventional simulations without biased potential constraint due to transition simulations on the scale of microseconds and longer. Nevertheless, we tried to obtain the mutually exclusive folding process from GL5/I27-S to GL5-ST/I27 models by using long time CMD simulation. Unfortunately, the process for the GL5/I27-S model did not succeed, which is consistent with the results of previous attempts by other researchers on large-scale conformational transition simulations. Targeted molecular dynamics (TMD) simulation is a method to observe large-scale conformational transition between two known end-point conformations of a molecule at ordinary temperature by applying a time-dependent with the help of a constraint. In TMD the root mean square deviation (RMSD) to a target structure as the offset parameter is constrained and continuously decreased during the simulation. The system is forced to find a path from its initial state to the final one. This time-dependent constraint in the TMD simulation introduces a bias which is minimal in the sense that only one degree of freedom is constrained [53]. The TMD simulation can generate plausible transition paths, and has been used in studies of allostery and a variety of transitions in large proteins [54]. A constraint energy term was added to the energy function proportional to the square of the difference which may be characterized as the mass-weighted RMSD of the current structure to the target structure in terms of atomic positions [55,56]. The functional form of the restraint energy can be written as

(1) $$E_{\mathrm{TMD}}=\frac{1}{2}kN{\left[RMSD(t)-RMSD_{0}(t)\right]}^{2}$$

where, *k* is the harmonic force constant per atom, *N* is the number of the restrained atoms, *RMSD*(*t*) is the root-mean-square deviation of the simulated structure at time *t* relative to the target structure, and *RMSD*_0_(*t*) is the prescribed target RMSD value at time *t* that decreases to zero linearly with time to drive the system from an initial structure to the target structure. To determine appropriate values of the harmonic force constant *k*, we tested the use of various *k* values for these TMD simulations (the data were depicted for the tested GL5/I27-S and GL5-ST/I27 models in Figure S6 of the Supplementary Materials), and chose *k* = 3.0 kcal/(mol·Å^2^) as the lowest harmonic force constant to apply to all heavy atoms of four models to bias the trajectories toward the target structure. Several TMD simulations starting from different initial unfolded structures for the transition of GL5/I27-S to GL5-ST/I27 models were performed to test the result consistency that is shown in Figure S7 of the Supplementary Materials. The corresponding result reflects that such TMD simulations are capable of driving significantly distinct starting structures to a non-distinguishable targeted one when simulations reach equilibrium. We tried to calculate the GL5/I27-S to GL5-ST/I27 process by forcing only folding atoms, and letting the other domain adapt freely. However, the simulation process takes such a long time that it could not be achieved in finite computation due to the fact that the freely adapted domain leads to too many random motions along the biased RMSD coordinates. The unfolding process could be achieved under a sufficiently long TMD simulation time. Moreover, steered molecular dynamics (SMD) can be utilized to simulate large-scale conformational transition by some other biased coordinates, such as the distance and angles etc. [57,58]. The computational details are provided in the Supplementary Materials.

## 5. Conclusions

Conventional molecular dynamics simulations were performed for the mutually exclusive folding protein of GL5/I27 to address the stable structure characteristics for the four mutually exclusive folding protein models with different unfolding degrees in the I27-guest or GL5-host domains. In the four models, the GL5/I27-S model involves the host domain GL5 and the secondary-structure unfolded guest domain I27-S, while the GL5/I27-ST model involves the host domain GL5 and the secondary/tertiary-structure extending guest domain I27-ST, and the other two GL5-S/I27 and GL5-ST/I27 models represent the secondary-structure unfolding and the secondary/tertiary-structure extending in the host domain GL5, respectively. Targeted molecular dynamics simulations were performed to address the mutually exclusive folding transition mechanisms from the two starting GL5/I27-S and GL5/I27-ST models with secondary-structure unfolding and secondary/tertiary-structure extending in the I27-guest domain to the two target GL5-ST/I27 and GL5-S/I27 models with the secondary-structure unfolding and full extending in the GL5-host domain. The variations of the radius of gyration (*R*_g_) and the fraction of native contacts (*Q*) for both I27-guest and GL5-host domains along the simulations trajectories were analyzed to investigate the nature of the mutually exclusive folding mechanism. For the process of GL5/I27-S model with the secondary-structure unfolding of the I27-guest domain to GL5-ST/I27 model with the full extending of the GL5-host domain, the slow formation of secondary structure of the I27-guest domain with a small deviation of radius of gyration *R*_g_ (*ΔR*_g_) of 1.8 Å causes significant extending of the GL5-host domain with *ΔR*_g_ of 7.8 Å. However, for the process of the GL5/I27-ST model with the secondary/tertiary-structure extending of the I27-guest domain to the GL5-ST/I27 model, the rapid decline of the I27-guest domain size by the decrease of *R*_g_ values from 18.9 Å to 12.6 Å induces insignificant extending of the GL5-host domain due to primary shrinking of the tertiary structure in the I27-guest domain. Then, the rapid extending of the GL5-host domain occurs along with further decrease of the I27-guest domain size. The results indicate that the folding of only the secondary-structure induces significantly full extending of the native GL5-host domain, and provides the main driving force for “tug-of-war” event of mutually exclusive folding/unfolding between the I27-guest and GL5-host domains. The variation of the fractions of native contacts *Q* for these processes also supports similar results discussed from the variation of *R*_g_ values. Through the investigation of the process of GL5/I27-S to GL5-S/I27, a special structure with both host and guest domains being folded at the same time as an intermediate was found, which was suggested by the experiment. The correlation analysis for some conformations predicts the structural characteristics of the native guest and host domains, and the mutually exclusive communication between the formation of the β sheets of the I27-guest domain and the extending of the GL5-host domain during the transition processes. The mutually exclusive folding principle is a special bio-function and could be used in some fields. For example, it may be expanded to construct a biosensor, or as a driving force to engineer self-assembling protein hydrogels.

## Figures and Tables

**Table 1:** Occupancies (%) of hydrogen bonds of the GL5-host domain for the GL5/I27-S, GL5-ST/I27 states and I, II, III, IV conformations. N–H···O represents the hydrogen bond between the N-H group and O atom.

	Hydrogen Bond	GL5/I27-S	I	II	III	IV	GL5-ST/I27
β1	(39)N–H···O(34,37)	99.84	96.6	86.12	–	–	–
	(33)N–H···O(29)	94.32	93.56	89.96	–	–	–
	(31)N–H···O(27)	99.8	95.64	93.68	–	–	–
	(29)N–H···O(25)	96.92	63.48	99.96	84	–	–
	(34)N–H···O(30)	99.72	97.76	96.48	57.12	–	–
	(26)N–H···O(22)	92.88	94.28	97.52	65.56	–	–
	(30)N–H···O(26)	99.48	78.8	88.68	46.36	–	–
	(35)N–H···O(31)	87.08	72.64	58.24	89.16	–	–
	(27)N–H···O(23)	99.28	96.68	91.88	75.36	–	–
	(32)N–H···O(28)	88	89.76	97.88	–	–	–
	(38)N–H···O(35)	79.92	28.92	25.6	–	–	–
	(28)N–H···O(24)	89.72	54.36	86.36	59.32	–	–
	(25)N–H···O(22)	45.12	53.76	28.72	–	–	–
β1–β2	(14)N–H···O(7)	50.24	52.68	24.56	–	–	–
	(20)N–H···O(1)	98	90.16	–	–	–	–
	(1)N–H···O(20)	45.64	39	–	–	–	–
	(9)N–H···O(12)	98.64	87.92	40.68	–	–	–
	(3)N–H···O(18)	98.64	98.52	68.44	–	–	–
	(18)N–H···O(3)	97.44	97.16	85.2	–	–	–
β1–β4	(10)N–H···OE1(152)	54.08	57.72	24.88	–	–	–
	(152)N–H···O(8)	90.48	–	–	–	–	–
	(148)N–H···O(4)	99.16	37.32	30.48	–	–	–
	(6)N–H···O(148)	98.32	99.76	99.48	–	–	–
	(150)N–H···O(6)	99.88	100	100	99.96	–	–
	(8)N–H···O(150)	59.68	79.04	48.72	55.44	–	–
β3–β4	(138)N–H···O(151)	98.52	–	–	–	–	–
	(151)N–H···O(138)	97	–	–	–	–	–
	(140)N–H···O(149)	97.92	94.24	40.72	–	–	–
	(142)N–H···O(147)	96.12	88.28	92.52	–	–	–

**Table 2:** Occupancies (%) of hydrogen bonds of I27-guest domain for the GL5/I27-S, GL5-ST/I27 states and I, II, III, IV conformations. N–H···O represents the hydrogen bond between the N-H group and O atom.

	Hydrogen Bond	GL5/I27-S	I	II	III	IV	GL5-ST/I27
βA-βG	(55)N–H···O(127)	–	–	–	–	75.96	99.64
	(57)N–H···O(129)	–	–	–	–	47.44	99.4
	(129)N–H···O(55)	–	–	–	–	64.96	91.6
	(123)N–H···O(43,44)	–	–	–	83.96	94.84	96.6
βA-βB	(49,50)N–H···O(68,69)	88.08	76.96	97.08	81.36	98.96	97.76
	(68)N–H···O(50)	–	–	–	–	–	97
	(61)N–H···O(58)	–	–	–	–	81.36	70.28
βB-βE	(63)N–H···O(104)	–	–	–	–	98.16	98.52
	(65)N-H···O(102)	–	–	–	–	90.56	99.36
	(100)N–H···O(66,67)	–	–	–	–	99.56	97.12
	(67,69)N–H···O(98,100)	–	–	68.08	95.72	95.92	98.36
	(107)N–H···O(61)	–	–	–	–	–	99.64
βE-βD	(111)N–H···O(108,133)	–	67.16	97.84	96.32	97.04	99.4
	(92)N–H···O(103)	–	–	–	–	90.04	99.68
	(103)N–H···O(92)	–	–	–	–	90.48	99.92
	(101)N–H···O(94)	–	–	–	–	63.48	99.84
	(96)N–H···O(93,99)	–	55.6	62.92	76.48	93.56	96.84
	(105)N–H···O(90)	–	–	–	–	71.04	84.24
βC-βD	(80)N–H···O(83)	–	44.08	61.28	78.28	85.84	81.68
	(90,91)N–H···O(87,88)	–	–	–	–	53.88	84.72
βC-βF	(77)N–H···O(118)	–	–	–	–	–	91.84
	(118)N–H···O(77)	–	–	–	–	–	98.84
	(117)N–H···O(80)	–	65.32	66.8	85.08	69.16	96.08
	(79)N–H···O(116)	–	–	–	51.16	69.16	96.08
	(116)N–H···O(79)	–	–	–	–	–	99.92
βF-βG	(128)N–H···O(113)	–	–	–	–	–	96.4
	(113)N–H···O(128)	–	–	–	–	–	98.56
	(126)N–H···O(114,115)	–	–	–	–	50.28	98.52
	(116,117)N–H···O(124)	–	–	–	–	69.4	99.68
	(124)N–H···O(117)	–	–	–	–	–	99.08
	(122)N–H···O(119)	–	33.04	96.84	82.72	94.96	96.96

## Supplementary Materials

Supplementary materials can be found at www.mdpi.com/1422-0067/17/11/1962/s1.
