# Supplementary material for: Influence of Secondary-Structure Folding on the Mutually Exclusive Folding Process of GL5/I27 Protein: Evidence from Molecular Dynamics Simulations
Source: Int J Mol Sci. 2016 Nov 23;17(11):1962. doi: 10.3390/ijms17111962 (PMC5133956; doi:10.3390/ijms17111962)
Supplement: Supplementary file 1 [file ijms-17-01962-s001.pdf]

Supplementary Materials: Influence of Secondary-Structure Folding on the Mutually Exclusive Folding Process of GL5/I27 Protein: Evidence from Molecular Dynamics Simulations

Qing Wang, Yan Wang and Guangju Chen

1. Details of Structure Construction

The details of the model construction for the GL5/I27-S and GL5/I27-ST models were described as follows: the folded coordinates of Met1–Val39 and Asp136–Gly152 sequences of the folded GL5-host domain and the folded coordinates of Leu45-Leu133 sequences of the I27-guest domain were taken from the NMR structures of the GB1 protein (PDB:2GB1) and the I27 protein (PDB:1TIT), respectively. These coordinates were loaded in the Discovery Studio visualizer (http://accelrys.com/) by adding the additional residues of Gly40–Gly44 and the linker of Leu134–Gly135 using "Insert after" option of "Build and Edit Protein" menu, and were positioned manually close to each other. The complex folded coordinates of GL5/I27 protein were generated with four inserted sites by using "Save" option in the Discovery Studio visualizer. The unfolded coordinates of I27-guest domain in the complex GL5/I27 protein were built through using repeatedly the "Build Loop" option of "Build" menu on several β strands in the Swiss-PdbViewer (also known as DeepView, http://spdbv.vital-it.ch/). Further, the unfolding sizes of the secondary-structure and the secondary/tertiary-structure for such complex coordinates were manually adjusted in the Discovery Studio visualizer. Finally, the two constructed GL5/I27-S and GL5/I27-ST models were fully minimized by using AMBER 9 package to obtain the appropriate joined coordinates of the folded GL5-host and unfolded I27-guest domains with the secondary-structure unfolding and the secondary/tertiary-structure extending. Similarly, the other two models of GL5-S/I27 and GL5-ST/I27 corresponding to the secondary-structure unfolding and the full extending in the GL5-host domain were built by using the same method.

2. Conventional Molecular Dynamics Simulation Protocols

All CMD simulations for these built models were carried out using the AMBER 9 package [1] and ff03 all atom force field parameters [2–4]. The protocol for all CMD simulations was described as follows: (1) the systems were energetically minimized to remove unfavorable contacts. Four cycles of minimizations were performed with 2500 steps of each minimization and harmonic restraints on the GL5/I27-S, GL5/I27-ST, GL5-S/I27 and GL5-ST/I27 models from 100, 75, 50 to 25 kcal/(mol·Å^2), which means that the restraints were relaxed stepwise by 25 kcal/(mol·Å^2) per cycle. The fifth cycle consists of 5000 steps of unrestrained minimization before the heating process. The cutoff distance used for the non-bonded interactions was 10 Å. The SHAKE algorithm [5] was used to restrain the bonds containing hydrogen atoms; (2) each energy-minimized structure was heated over 120 ps from 0 to 300 K (with a temperature coupling of 0.2 ps, while the positions of the GL5/I27-S, GL5/I27-ST, GL5-S/I27, and GL5-ST/I27 models were restrained with a small value of 25 kcal/(mol·Å^2). Constant volume was maintained during the processes; (3) the unrestrained equilibration of 200 ps with constant pressure and temperature conditions was carried out for each system. The temperature and pressure were allowed to fluctuate around 300 K and 1 bar, respectively, with the corresponding coupling of 0.2 ps. A Langevin thermostat (NTT = 3) was used for the temperature regulation of each system. For each simulation, an integration step of 2 fs was used; and (4) finally, conventional molecular dynamics (CMD) runs of 50 ns for GL5/I27-S, GL5/I27-ST, GL5-S/I27, and GL5-ST/I27 models were carried out, respectively, by following the same protocol.

3. Targeted Molecular Dynamics Simulation Protocols

In the targeted molecular dynamics simulations [6,7], a time-dependent constraint force was applied onto the positions of all the heavy atoms of the GL5/I27-S, GL5/I27-ST, GL5-S/I27 and GL5-ST/I27 models at each time-step to bias the trajectory toward the corresponding target structure. To obtain the appropriate small force constant *k*, four 10-ns independent targeted molecular dynamics (TMD) simulations (including the last 2 ns of equilibration) were performed using different force constants of 0.5, 1.0, 2.0, 3.0, and 4.0 kcal/(mol·Å^2), respectively. As shown in Figure S5 for the conversion of GL5/I27-S to GL5-ST/I27, three out of the four TMD simulations reached a *RMSD* value of <0.5 Å extracting from the backbone atoms of two GL5/I27-S and GL5-ST/I27 models. To achieve the satisfied transition process of the initial structure GL5/I27-S to the target structure GL5-ST/I27, k = 3.0 kcal/(mol·Å^2) was chosen as the lowest harmonic force constant to apply onto all the heavy atoms of four models to bias the trajectories toward the target structure. All the other atoms in the two GL5/I27-S and GL5-ST/I27 models were assumed to move freely and to rearrange at their convenience to let the proteins reach their equilibrium under this constraint. The weighted RMSDs along the TMD trajectories from four, 10-ns independent simulations showed that each process could follow the same conformational transition pathway, which indicated that the external forces and initial velocities did not affect the transition pathway. Therefore, the TMD simulations with the 8 ns simulation time followed by 2 ns of equilibration with an integration step of 2 ps were performed for these four mutually exclusive processes.

4. Calculations of the Radius of Gyration R_g, the Fraction of the Native Contacts *Q*, Hydrogen Bond, and the Correlation of Atomic Motions

The radius of gyration R_g as a global measure was calculated to monitor the "size" of the protein, which is defined as [8–10]:

$$R_{\mathrm{g}}^2 = \frac{1}{N}\sum_j \left| r_j - \langle r \rangle \right|^2 \tag{1}$$

where r_j is the position vector of the *j*th atom, *N* is the total number of atoms, $\langle r \rangle = \sum_j r_j / N$ is the mean of the position vectors, and $R_{\mathrm{g}} = \sqrt{R_{\mathrm{g}}^2}$. The fraction of native contacts *Q* defined as the number of native residue–residue contacts in the predicted complex divided by the number of native contacts in the target was used as order parameter for the folding degrees (Q = 0 at the fully unfolded state and Q = 1 at the folded state) [11–13]. In recent years, these two parameters have been shown to be a key determinant of transition state structure and the mechanism of protein folding [14,15]. A "hydrogen bond" is defined as a distance of less than 3.5 Å between a hydrogen atom attached to either an oxygen or a nitrogen atom and an acceptor atom, and as an angle formed by a donor, a hydrogen atom and an acceptor being larger than 120° with the corresponding occupancy of ≥30%. To compare the amount of the total possible hydrogen bonds in the various transition conformations, a relative percentage of total hydrogen bond occupancies for a conformation was calculated by defining the relative percentage equal to one hundred percent when the all possible hydrogen bonds occupy each snapshot in the reactant during the mutually exclusive process. These parameter analyses in the present work were calculated by using the PTRAJ module of the AMBER 9 program.

The dynamic feature of a protein and the extent of correlation of motions in the different regions of a protein were assessed via the calculation of cross-correlation coefficients, C(*i*, *j*), given as follows:

$$C(i,j) = \langle \Delta r_i \times \Delta r_j \rangle \Big/ \left(\langle \Delta r_i^{\,2} \rangle \langle \Delta r_j^{\,2} \rangle \right)^{1/2} \tag{2}$$

In the equation, Δr_i and Δr_j are the displacement vectors for Cα atoms of residues *i* and *j*, respectively, and the angle brackets denote the ensemble averages [4]. The correlation coefficients

were averaged over the regions of protein, and the resulted cross-correlation coefficients are presented in the form of a two-dimensional graph.

The correlation of motions analyses in the present work were calculated by using the PTRAJ module of the AMBER 9 program.

Table S1. Occupancies (%) of hydrogen bonds of the GL5-host domain for the GL5/I27-ST, GL5-ST/I27 states and I′, II′, III′, IV′, V′ conformations. N–H···O represents the hydrogen bond between the N-H group and O atom.

	Hydrogen Bond	GL5/I27-ST	I′	II′	III′	IV′	V′	GL5-ST/I27
α1	(39)N–H···O(34)	99.96	99.92	99.84	99.84	99.28	–	–
	(33)N–H···O(29)	92.12	94.12	94.84	92.88	70.68	–	–
	(31)N–H···O(27)	99.84	99.64	99.68	72.8	–	–	–
	(29)N–H···O(25)	79.44	91.44	97	99.96	99.92	–	–
	(34)N–H···O(30)	99.88	99.88	99.56	95.56	68.24	–	–
	(26)N–H···O(22)	94.64	88.6	96	98.28	99.44	–	–
	(30)N–H···O(26)	97	98.36	99.32	85.04	–	–	–
	(35)N–H···O(31)	80.44	65.08	92.92	82.24	44.84	–	–
	(27)N–H···O(23)	99.32	99.72	99.64	84.04	68.84	–	–
	(32)N–H···O(28)	83.28	73.4	95.56	72.2	71.36	–	–
	(38)N–H···O(35)	75.12	60.84	81.4	72.32	59.32	–	–
	(28)N–H···O(24)	57.92	90.92	81.68	76.92	42.36	–	–
	(25)N–H···O(22)	53.08	48.72	48.4	29.56	–	–	–
β1–β2	(14)N–H···O(7)	98.32	99.48	99.72	98.96	–	–	–
	(20)N–H···O(1)	93.92	97.12	98.76	77.72	–	–	–
	(1)N–H···O(20)	62.16	41.64	40	–	–	–	–
	(9)N–H···O(12)	98.56	94.8	35.92	42.48	–	–	–
	(3)N–H···O(18)	99.24	99.8	97.32	99.04	–	–	–
	(18)N–H···O(3)	97.32	98.84	98.4	95	–	–	–
β1–β4	(10)N–H···OE2(152)	12.76	45.48	89.44	45.16	50.12	–	–
	(152)N–H···O(8)	88.96	93.16	32.4	–	–	–	–
	(148)N–H···O(4)	100	99.88	99.8	–	–	–	–
	(6)N–H···O(148)	98.4	99.72	99.76	91.68	–	–	–
	(150)N–H···O(6)	83.88	99.96	99.96	99.56	99.92	–	–
	(8)N–H···O(150)	28.48	79.8	81.44	64.88	–	–	–
β3–β4	(138)N–H···O(151)	99.24	98.88	98.4	–	–	–	–
	(151)N–H···O(138)	95.48	97.84	98.32	–	–	–	–
	(140)N–H···O(149)	97.8	98.96	98	30.96	–	–	–
	(142)N–H···O(147)	93.04	95.28	95.12	92.36	–	–	–

Table S2. Occupancies (%) of hydrogen bonds of the I27-guest domain for the GL5/I27-ST, GL5-ST/I27 states and I′, II′, III′, IV′, V′ conformations. N–H···O represents the hydrogen bond between the N-H group and O atom.

	Hydrogen Bond	GL5/I27-ST	I′	II′	III′	IV′	V′	GL5-ST/I27
βA–βG	(55)N–H···O(64,127)	–	–	96	98.56	99.96	84.56	99.64
	(129)N–H···O(55)	–	–	–	–	–	–	91.6
	(121,123)N–H···O(43,44)	–	–	–	–	46.84	–	96.6
	(43,44)N–H···O(121,123)	–	–	–	43.56	64.52	–	84.72
βA–βB	(57)N–H···O(62,129)	–	–	99.92	99	100	72.32	99.4
	(49,50)N–H···OE2(68)	–	–	29.64	59.64	38.56	95.4	97.76
	(68)N–H···O(50)	–	–	–	–	–	99.48	97
	(61)N–H···O(58)	–	–	–	–	–	–	70.28
	(70)N–H···O(47)	–	–	–	–	–	90.4	97.96
βB–βE	(63)N–H···O(104)	–	–	–	91.04	94.96	99.24	98.52
	(65)N-H···O(102)	–	–	–	–	—	50.64	98.04
	(100)N–H···O(66,67)	–	–	–	–	–	97.64	97.12
	(67,69)N–H···O(98,100)	–	–	–	–	–	97.32	98
	(105,107)N–H···O(61)	–	–	–	–	43.32	–	99.64
βE–βD	(111)N–H···O(108)	–	–	–	–	–	99.56	99.4
	(92)N–H···O(103)	–	–	–	–	–	–	99.68
	(103)N–H···O(92)	–	–	–	–	–	–	99.92
	(101)N–H···O(94)	–	–	–	–	–	–	99.84
	(96)N–H···O(99)	–	–	–	–	–	89.72	96.84
	(105)N–H···O(90)	–	–	–	–	–	–	84.24
βC–βD	(80)N–H···O(83)	–	–	–	–	85.76	99.28	81.68
	(90,91)N–H···O(87,88)	–	–	–	–	51.6	53.6	84.72
βC–βF	(77)N–H···O(118)	–	–	–	–	–	98.96	91.84
	(118)N–H···O(77)	–	–	–	–	–	97.32	98.84
	(79)N–H···O(116)	–	–	–	–	–	97.96	96.08
	(116)N–H···O(79)	–	–	–	–	–	99.56	99.92
βF–βG	(127,128)N–H···O(112,113)	–	–	–	–	60.52	96.68	96.4
	(113)N–H···O(128)	–	–	–	–	–	90.04	98.56
	(126)N–H···O(115)	–	–	–	–	–	98.96	98.52
	(117,118)N–H···O(124)	–	–	–	–	41.48	97.56	99.68
	(124)N–H···O(117,118)	–	–	–	–	–	94.68	99.08
	(122)N–H···O(119)	–	–	–	–	71.8	81.04	96.96

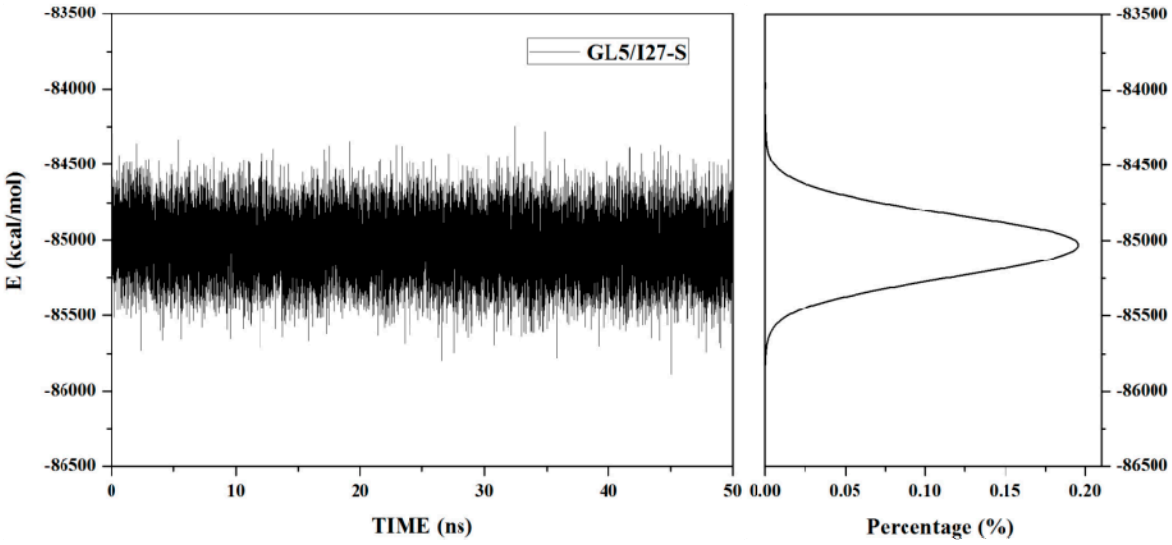

(**a**)

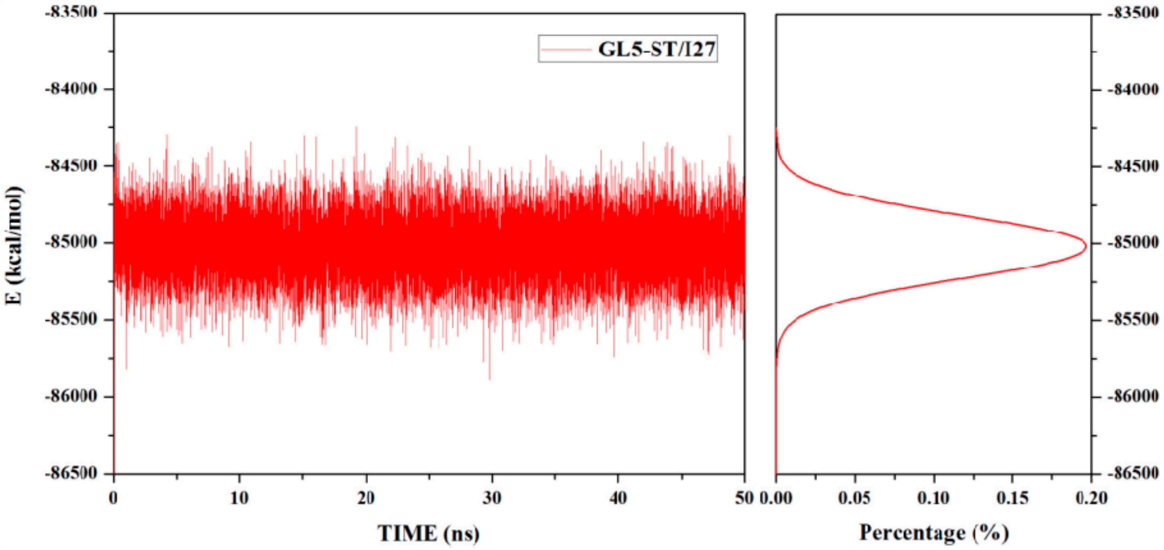

(**b**)

Figure S1. Stability conformational energy (kcal·mol^{-1}) and integrated distributions for (**a**) the GL5/I27-S model (black) and (**b**) the GL5-ST/I27 model (red).

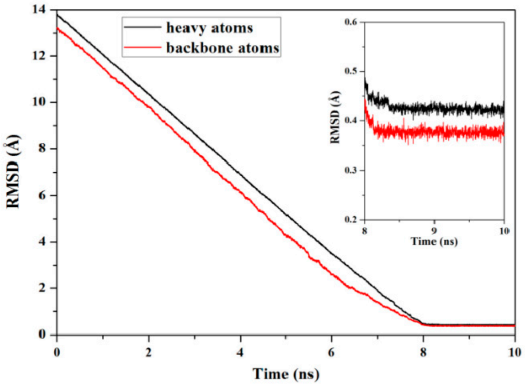

(**a**)

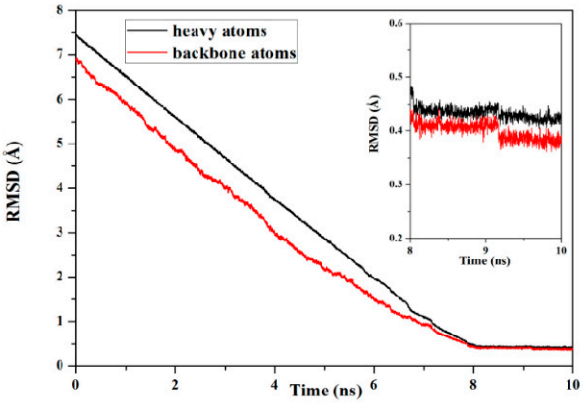

(**b**)

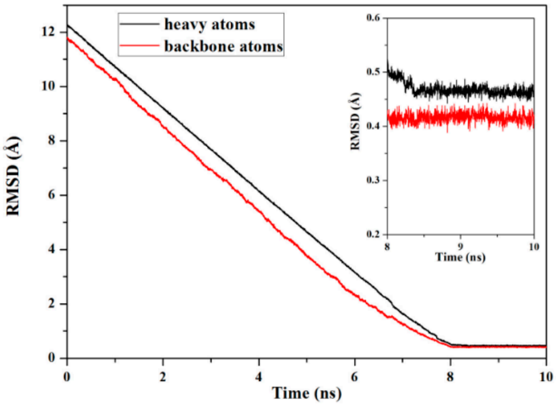

(**c**)

Figure S2. Root mean square deviation (*RMSD*) values all backbone atoms and all heavy atoms of the whole protein from the targeted molecular dynamics (TMD) simulations of transitions of (**a**) GL5/I27-ST to GL5-ST/I27; (**b**) GL5/I27-S to GL5-S/I27; (**c**) GL5/I27-ST to GL5-S/I27.

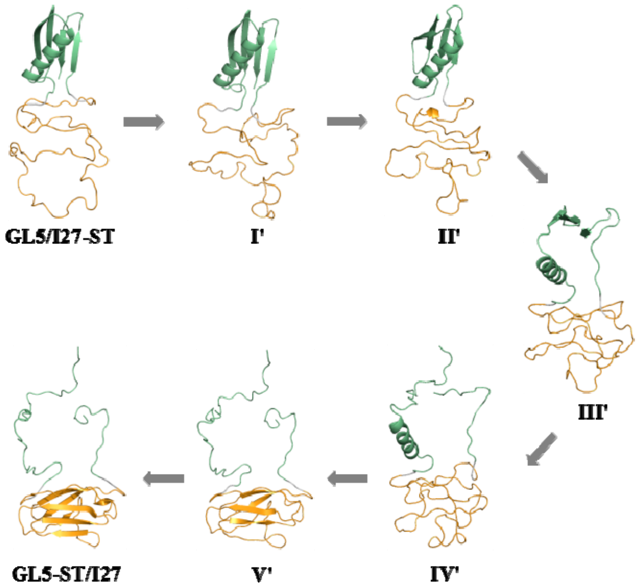

Figure S3. The three-dimensional average structures for GL5/I27-ST, GL5-ST/I27 states and I′, II′, III′, IV′, V′ conformations for the transition of GL5/I27-ST to GL5-ST/I27. The components of I27-guest domains, GL5-host domains and linkers are colored in orange, olive and gray, respectively.

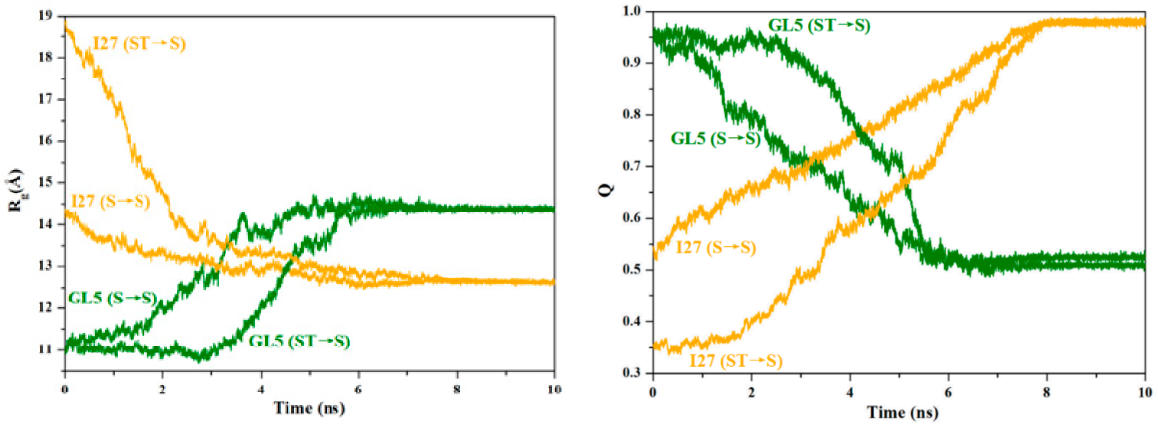

(**a**) (**b**)

Figure S4. Variations for (**a**) the radius of gyration (R_g) values and (**b**) the fractions of native contacts (Q) values of I27-guest (orange) and GL5-host domains (olive) for GL5/I27-S and GL5/I27-ST models respectively to GL5-S/I27 model (i.e., S→S and ST→S).

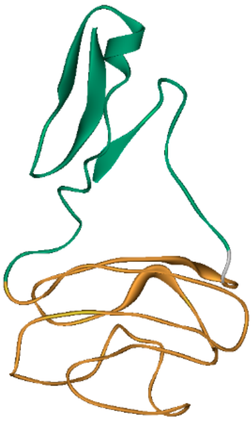

Figure S5. The three-dimensional average structure of the intermediate with both I27-guest and GL5-host domains being folded at the same time during the process of GL5/I27-S to GL5-S/I27. The components of I27-guest domains, GL5-host domains and linkers are colored in orange, olive and gray, respectively.

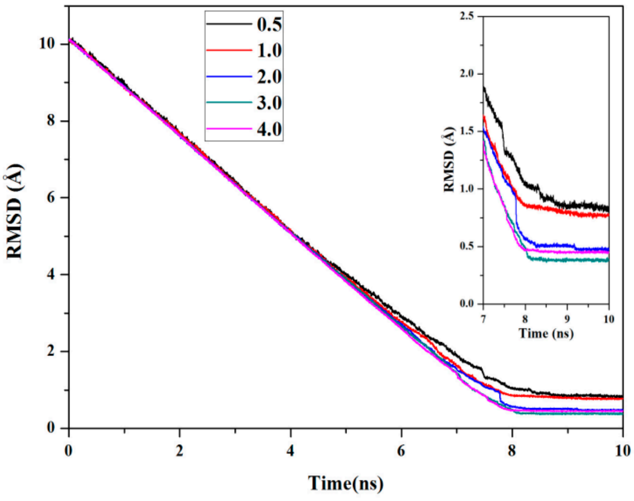

Figure S6. The root mean square deviation (*RMSD*) values of all backbone atoms of the whole protein for the transition of GL5/I27-S to GL5-ST/I27 using varying force constants *k*, in kcal/(mol·Å^2), shown in the inset.

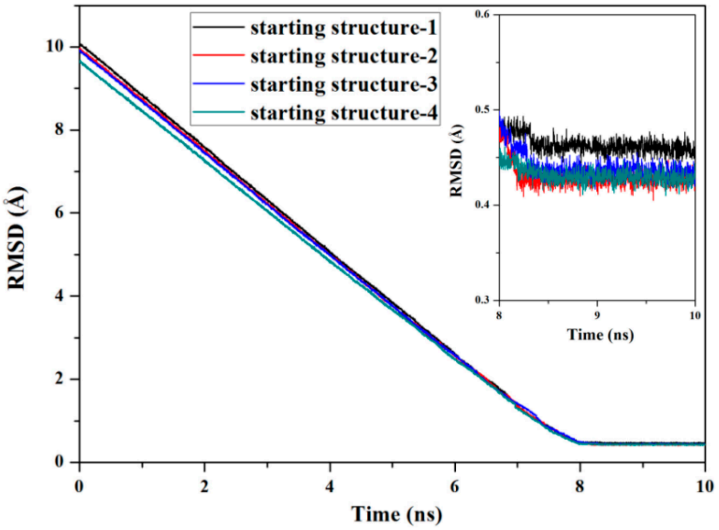

Figure S7. The root mean square deviation (*RMSD*) values of all heavy atoms of the whole protein from four TMD simulations starting from different initial unfolded structures for the transition of GL5/I27-S to GL5-ST/I27.

References

1. Case, D.; Darden, T.; Cheatham, T., III; Simmerling, C.; Wang, J.; Duke, R.; Luo, R.; Merz, K.; Pearlman, D.; Crowley, M.; et al. *Amber 9*; University of California: San Francisco, CA, USA, 2006.
2. Duan, Y.; Wu, C.; Chowdhury, S.; Lee, M.C.; Xiong, G.; Zhang, W.; Yang, R.; Cieplak, P.; Luo, R.; Lee, T. A point-charge force field for molecular mechanics simulations of proteins based on condensed-phase quantum mechanical calculations. *J. Comput. Chem.* **2003**, *24*, 1999–2012.
3. Lee, M.C.; Duan, Y. Distinguish protein decoys by using a scoring function based on a new amber force field, short molecular dynamics simulations, and the generalized born solvent model. *Proteins Struct. Funct. Bioinform.* **2004**, *55*, 620–634.
4. Wang, J.; Wolf, R.M.; Caldwell, J.W.; Kollman, P.A.; Case, D.A. Development and testing of a general amber force field. *J. Comput. Chem.* **2004**, *25*, 1157–1174.
5. Miyamoto, S.; Kollman, P.A. Settle: An analytical version of the shake and rattle algorithm for rigid water models. *J. Comput. Chem.* **1992**, *13*, 952–962.
6. Schlitter, J.; Engels, M.; Krüger, P. Targeted molecular dynamics: A new approach for searching pathways of conformational transitions. *J. Mol. Graph. Model.* **1994**, *12*, 84–89.
7. Schlitter, J.; Engels, M.; Krüger, P.; Jacoby, E.; Wollmer, A. Targeted molecular dynamics simulation of conformational change-application to the T ↔ R transition in insulin. *Mol. Simul.* **1993**, *10*, 291–308.
8. Knott, M.; Chan, H.S. Criteria for downhill protein folding: Calorimetry, chevron plot, kinetic relaxation, and single-molecule radius of gyration in chain models with subdued degrees of cooperativity. *Proteins Struct. Funct. Bioinform.* **2006**, *65*, 373–391.
9. Kuwajima, K.; Hiraoka, Y.; Ikeguchi, M.; Sugai, S. Comparison of the transient folding intermediates in lysozyme and .alpha.-lactalbumin. *Biochemistry* **1985**, *24*, 874–881.
10. Mark, A.E.; van Gunsteren, W.F. Simulation of the thermal denaturation of hen egg white lysozyme: Trapping the molten globule state. *Biochemistry* **1992**, *31*, 7745–7748.
11. Duan, Y.; Kollman, P.A. Pathways to a protein folding intermediate observed in a 1-microsecond simulation in aqueous solution. *Science* **1998**, *282*, 740–744.
12. Ferrara, P.; Caflisch, A. Native topology or specific interactions: What is more important for protein folding? *J. Mol. Biol.* **2001**, *306*, 837–850.
13. Méndez, R.; Leplae, R.; de Maria, L.; Wodak, S.J. Assessment of blind predictions of protein–protein interactions: Current status of docking methods. *Proteins Struct. Funct. Bioinform.* **2003**, *52*, 51–67.

14. Karanicolas, J.; Brooks, C.L. The origins of asymmetry in the folding transition states of protein l and protein g. *Protein Sci.* **2002**, *11*, 2351–2361.
15. Shea, J.-E.; Brooks, C.L., III. From folding theories to folding proteins: A review and assessment of simulation studies of protein folding and unfolding. *Annu. Rev. Phys. Chem.* **2001**, *52*, 499–535.
